# Supplementary material for: Brassinosteroids Regulate the Water Deficit and Latex Yield of Rubber Trees
Source: Int J Mol Sci. 2023 Aug 16;24(16):12857. doi: 10.3390/ijms241612857 (PMC10454136; doi:10.3390/ijms241612857)

Figure S1. Effects of exogenous BR application on photosynthesis in rubber tree leaves under water deficit stress.

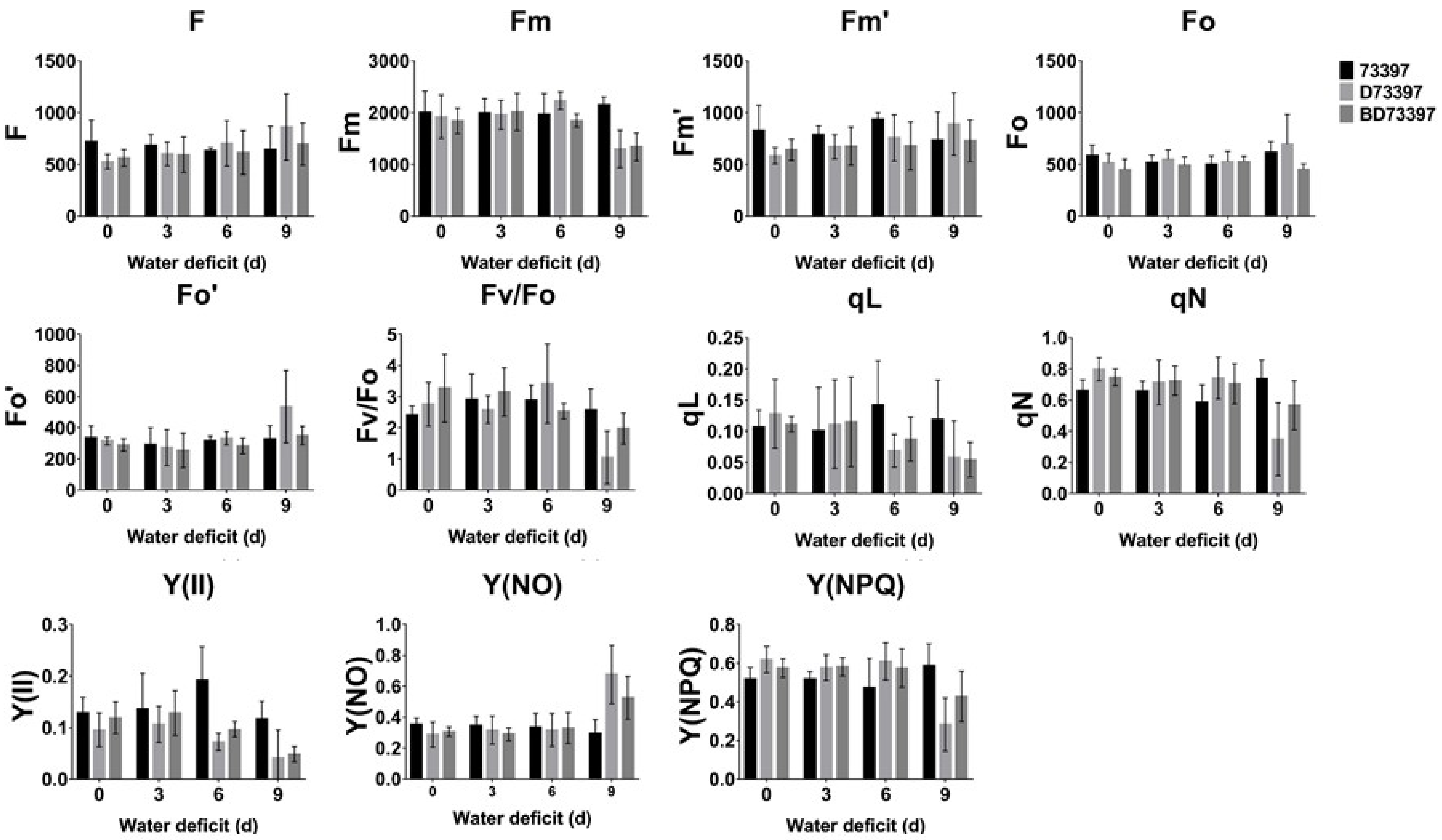

Supplement: Supplementary file 1 [file ijms-24-12857-s001.zip › Supplementary/Figure S1.pdf]
