# Supplementary material for: Brassinosteroids Regulate the Water Deficit and Latex Yield of Rubber Trees
Source: Int J Mol Sci. 2023 Aug 16;24(16):12857. doi: 10.3390/ijms241612857 (PMC10454136; doi:10.3390/ijms241612857)
Supplement: Supplementary file 1 [file ijms-24-12857-s001.zip › Supplementary/Table S1.pdf]

**Table S1**. Primers used in this study

| Gene | Forward | Reverse |
|---|---|---|
| *HbActin* | GATGTGGATATCAGGAAGGA | CATACTGCTTGGAGCAAGA |
| *HbAPX* | CCAACTGACACCGTTCTT | CAGCACCATCCTCTACATC |
| *HbCAT* | GGTATTGTGGTTCCTGGTAT | ATGGTGATTGTTGTGATGAG |
| *HbMnSOD* | TGTGCTGTAATGTTGACCTA | GTTCACCTGTAAGTAGTATGC |
| *HbPOD* | CAGGGAGGAGGGACAGCAAAGA | GTTCCTTCAGCGTCAATGCCAATG |
| *HbRbsS* | GCCAAGGAAGTTGAATACC | CCAGTAACGACCATCATAGT |
| *HbFPS* | TTGCTTTGGTGATCCCGAGAC | AGGACTTCAACACTGCTTGCAC |
| *HbHMGR* | GAATTGGCAGGTAAGCGGGTG | GGTGTCAACTTCTGTGAGATAG |
| *HbGGPPS* | CAAGCTACCAGATCCAGATC | GGGAGGTCATCATGGATAAG |
| *HbSRPP* | TGGAGATGAGGCATCATAATCTGCA | TATTCCACATCCAAAACACACCACC |
| *HbHRT* | GCAAATGCAACTGGAAGCGG | AGACGGGTCTCCCCAGAAGT |
| *HbREF* | ACCAAGAGACTTTCTAAGGTGCTA | CACTTCATCATCTTACTCAACTGG |
| *HbHEV* | GCAATTGCTGAGCAATGTGG | GTTGCTTATTGCTTGAGAAGC |
| *HbHGN* | CCTCAAGAACTACTGTCATGC | GGTGACCTGATAGAGGATAG |
| *HbCHI* | GTCCAGCTCTCATGTTGATG | GAGCTATACTGGCATGGTGG |
